# Chitooligosaccharides Derivatives Protect ARPE-19 Cells against Acrolein-Induced Oxidative Injury

**DOI:** 10.3390/md21030137

**Published:** 2023-02-22

**Authors:** Cheng Yang, Rongrong Yang, Ming Gu, Jiejie Hao, Shixin Wang, Chunxia Li

**Affiliations:** 1 Shandong Key Laboratory of Glycoscience and Glycotechnology, Key Laboratory of Marine Drugs of Ministry of Education, School of Medicine and Pharmacy, Ocean University of China, Qingdao 266003, China; 2 Laboratory for Marine Drugs and Bioproducts, Pilot National Laboratory for Marine Science and Technology (Qingdao), Qingdao 266237, China; 3 Laboratory of Marine Glycodrug Research and Development, Marine Biomedical Research Institute of Qingdao, Qingdao 266071, China

**Keywords:** chitosan oligosaccharide, *N*-acetylated chitosan oligosaccharide, ARPE-19, oxidative stress, Nrf2

## Abstract

Age-related macular degeneration (AMD) is the leading cause of vision loss among the elderly. The progression of AMD is closely related to oxidative stress in the retinal pigment epithelium (RPE). Here, a series of chitosan oligosaccharides (COSs) and *N*-acetylated derivatives (NACOSs) were prepared, and their protective effects on an acrolein-induced oxidative stress model of ARPE-19 were explored using the MTT assay. The results showed that COSs and NACOs alleviated APRE-19 cell damage induced by acrolein in a concentration-dependent manner. Among these, chitopentaose (COS–5) and its *N*-acetylated derivative (N–5) showed the best protective activity. Pretreatment with COS–5 or N–5 could reduce intracellular and mitochondrial reactive oxygen species (ROS) production induced by acrolein, increase mitochondrial membrane potential, GSH level, and the enzymatic activity of SOD and GSH-Px. Further study indicated that N–5 increased the level of nuclear Nrf2 and the expression of downstream antioxidant enzymes. This study revealed that COSs and NACOSs reduced the degeneration and apoptosis of retinal pigment epithelial cells by enhancing antioxidant capacity, suggesting that they have the potential to be developed into novel protective agents for AMD treatment and prevention.

## 1. Introduction

Age-related macular degeneration (AMD) is one of the main causes of irreversible central visual loss in the elderly worldwide [1,2]. In 2020, the number of AMD patients worldwide was 19.6 million, and this number was predicted to be 288 million in 2040 [1,3,4]. According to the symptoms, AMD can be divided into dry and wet forms with 80% and 20% prevalence, respectively [5]. Current treatments for wet AMD include laser therapy and VEGF antibody injection (such as Eylea) [6], while there is no preventive therapy for dry AMD [7]. Therefore, it is necessary to develop effective agents to prevent or cure dry AMD.

Numerous studies have indicated that AMD pathogenesis was related with chronic oxidative stress and the inflammation of retinal pigment epithelial (RPE) cells, which could lead to the eventual degeneration of the RPE [8,9,10,11]. Jin et al. [12] suggested that retinal pigment epithelium cell apoptosis was induced by ultraviolet and hydrogen peroxide via AMPK signaling. *Melissa officinalis* L. extracts and resveratrol were reported to improve cell viability and decrease reactive oxygen species (ROS) generation in RPE cells to prevent AMD [10,13]. These results demonstrated that the inhibition of RPE cell damage induced by ROS could prevent the process of AMD [14]. Therefore, antioxidation could be an effective strategy to protect RPE cells for the amelioration of early AMD.

Chitin is extracted mainly from the shells of crabs, shrimps and insects and is one of the most abundant natural biopolymers [15,16,17]. Chitosan oligosaccharides (COSs) are the degraded product of chitin or chitosan and consist of glucosamine linked by β-1,4-glycosidic bonds, possess various biological effects, including anti-inflammatory, antimicrobial, immunomodulatory, antioxidant, and anticancer activities [18,19,20,21]. Fang demonstrated that COS attenuated oxidative-stress related retinal degeneration in a dose-dependent manner in a rat model [22]. Xu found that COSs protected against Cu(II)-induced neurotoxicity in primary cortical neurons by interfering with an increase in intracellular reactive oxygen species (ROS) [23]. Our previous study indicated that peracetylated chitosan oligosaccharide (PACOs) pretreatment significantly reduced lactate dehydrogenase release and reactive oxygen species production in PC12 cells [24]. In addition, Guo’s group indicated that the antioxidant properties of chitosan were inversely related to its molecular weight (Mw) [25]. We performed a preliminary screening of structurally related compounds, and COSs and NACOs showed excellent antioxidant activity with the potential to prevent AMD. In the present study, we investigated the effect of a series of chitosan oligosaccharides and their *N*-acetylated derivatives on RPE cell damage and explored the possible mechanisms of action. The results showed that chitosan oligosaccharides had an excellent capacity for protecting RPE cells from acrolein-induced oxidative stress.

## 2. Results and Discussion

### 2.1. Characterization of Chitooligosaccharides and N-Acetylated Chitooligosaccharides

According to the previous method, chitooligosaccharides (COSs) and *N*-acetylated chitooligosaccharides (NACOs) were prepared via enzymatic hydrolysis [24] and acetylated modification [26].

**Figure 1:**
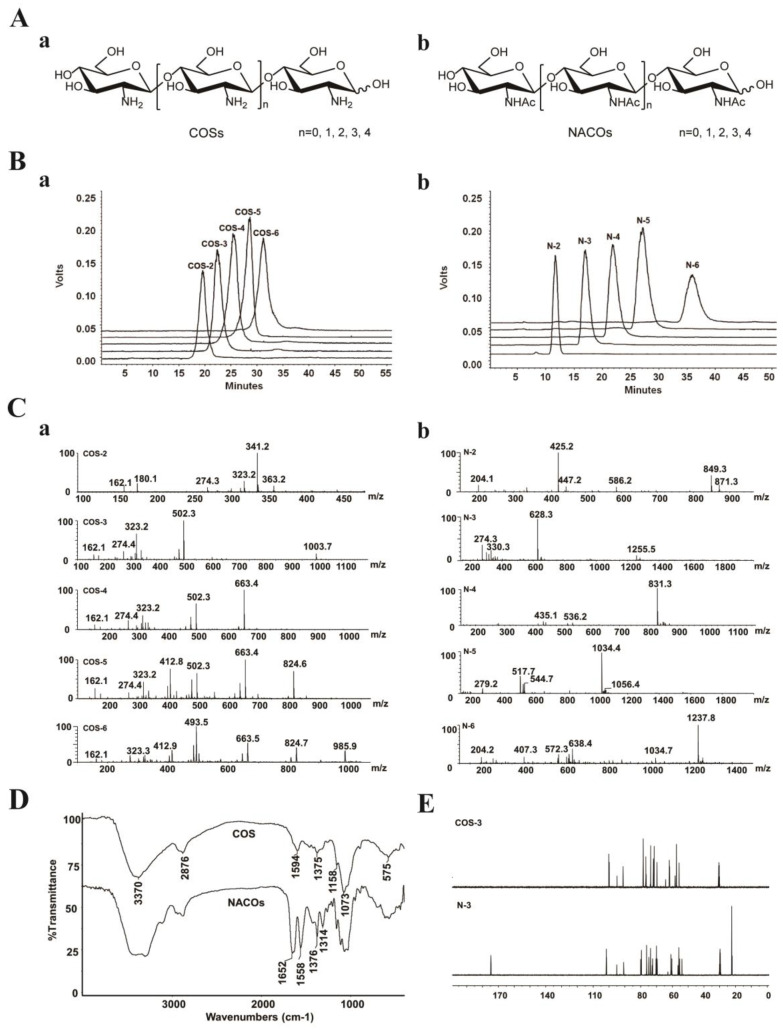
Characterization of COSs and NACOs. (**A**) Schematic structures of COSs (**a**) and NACOs (**b**). (**B**) HPLC chromatograms of COSs (**a**) and NACOs (**b**). (**C**) MS spectra of COSs (**a**) and NACOs (**b**). (**D**) IR spectra of COSs and NACOs. (**E**) ^13^C NMR (125 MHz, D_2_O) of COS–3 and N–3.

The crude products were isolated and purified to provide monomers with different degree of polymerization (Figure 1A). NACOs were purified by column chromatography using graphitized carbon black as the stationary phase and ethanol–water as the mobile phases. This purification method was simple and efficient with the elimination of the tedious operation process of desalting, compared with gel exclusion and ion exchange purification methods [27]. The purity of these compounds was analyzed via HPLC (LC-10AD, Shimadzu, Kyoto, Japan) [28,29]. As shown in Figure 1B, the purity of COS (COS–2~6) and NACO (N–2~6) monomers was above 95%.

The structures of COSs and NACOSs were characterized using a quadrupole time of flight (Q-TOF) mass spectrometer, and nuclear magnetic resonance (NMR) and Fourier-transform infrared spectroscopy (FT-IR) analysis (Figure 1C–E). The Q-TOF MS analysis (positive ion mode) of COSs and NACOs samples are shown in Figure 1C and Table 1.

For the FT-IR spectra (Figure 1D), the bands at 3370 cm^−1^, 2876 cm^−1^, and 1073 cm^−1^ were corresponded to stretching vibrations of O-H, C-H and C-O, respectively. The spectra of *N*-acetyl chitosan oligosaccharides showed the characteristic absorptions of 1649 cm^−1^, 1549 cm^−1^ and 1314 cm^−1^, which were attributed to amide I, II and III bands of amide, respectively [30]. Moreover, there was no 1735 cm^−1^ band (-C(=O)O-) in the *N*-acetylated chitosan oligosaccharide, indicating no acetylation on the OH groups of COSs.

The structures of COSs and NACOSs were also characterized via NMR. Taking COS–3 and N–3 as examples, the 13C NMR signals in spectra (Figure 1E) were assigned in Table 2. Compared to COS–3, acetyl signal peaks appeared in the 13C NMR spectrum of N–3, with 174.8 ppm attributed to C=O, and peaks at 22.6~22.3 ppm attributed to CH_3_ of acetyls.

### 2.2. Protective Effect of COSs and NACOs against Acrolein-Induced Cell Death

The cytotoxicity of COS and NACO monomers (DP 2, 3, 4, 5, 6) was tested via MTT assay in ARPE-19 cells. After 24 h of incubation with 1 mM COSs or NACOs, the MTT test showed that both COSs and NACOs exhibited no significant cytotoxicity (Figure S1A). In addition, the effect of COSs and NACOs on the viability of ARPE-19 cells was tested with different concentrations (200, 400, 800 μM). It showed that COSs and NACOs did not affect cell proliferation. (Figure S2).

Acrolein, a major component of the gas phase of cigarette smoke and also a product of lipid peroxidation in vivo, has been shown to be a mitochondrial toxicant related to mitochondrial dysfunction [31]. Therefore, acrolein-induced cellular oxidative mitochondrial dysfunction in retinal pigment epithelial (RPE) cells had been used as a cellular model to evaluate antioxidants and mitochondrial protecting agents [32,33,34]. Here, AREP-19 cells were pretreated with different concentrations of COSs or NACOs (200, 400 and 800 μM) for 48 h, and then treated with 75 μM acrolein for 24 h, and cell viability was measured using the MTT test.

**Figure 2:**
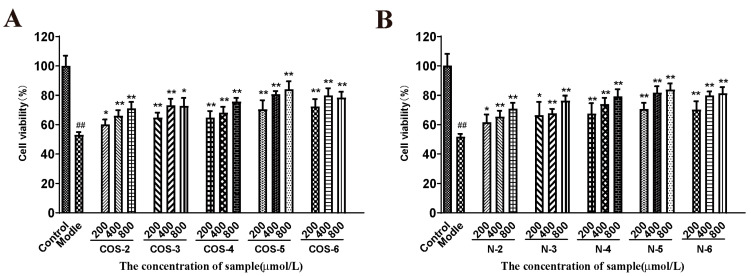
Protective effect of COSs and NACOs against acrolein-induced ARPE-19 cell death. The cells were pretreated with 200, 400, 800 μM COSs (**A**) or NACOs (**B**) for 48 h and then treated with 75 μM acrolein for an additional 24 h. Cell viability was analyzed using the MTT method. Values are mean ± SD of five separate experiments. ^##^ *p* < 0.01 vs. control (no acrolein, no COSs or NACOs); * *p* < 0.05, ** *p* < 0.01 vs. acrolein.

As shown in Figure 2, the cells exposed to 75 µM acrolein showed a significant decrease in cell viability (about 50%) compared to the untreated control group. However, after pretreatment with COSs or NACOs at 200, 400, and 800 μM for 48 h before acrolein exposure, the cell viability increased significantly. Furthermore, COSs and NACOs exhibited similar protective activity which was dose-dependent. In addition, we also prepared peracetylated chitosan oligosaccharides (PACOs) [24], but PACOs had a certain cytotoxicity to ARPE-19 (Figure S1B). Glucosamine pentamer (COS–5) and *N*-acetylated chitopentaose (N–5) showed the highest protective activity, which indicated that the pentaose skeleton may be the suitable structure for binding to the receptors or targets, such as heparin core pentasaccharide, for anticoagulant activity [35].

### 2.3. Protective Effect of COS–5 and N–5 against Acrolein-Induced Oxidative Stress

**Figure 3:**
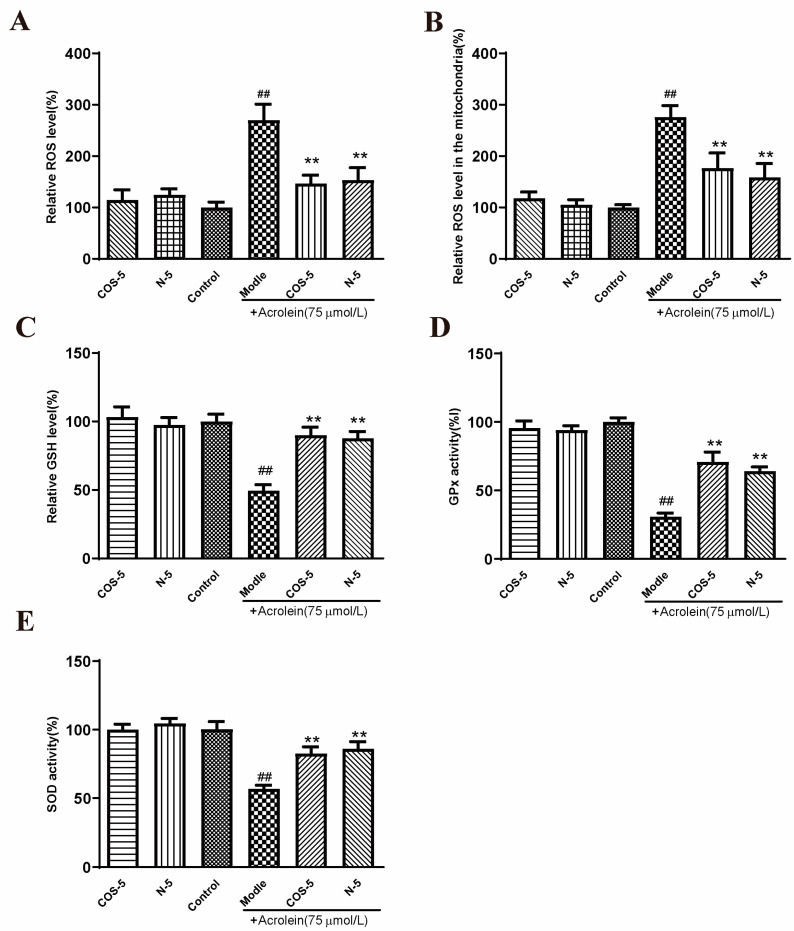
COS–5 and N–5 against acrolein-induced oxidative stress. ARPE-19 cells were treated with 400 μM COS–5 or N–5 for 48 h and then treated with acrolein for an additional 24 h. Cellular ROS generation (**A**), ROS level in mitochondria (**B**), GSH level (**C**), GPx (**D**) and SOD activities (**E**). The data expressed as ratio relative to controls. Values are mean ± SD of five separate experiments. ^##^ *p* < 0.01 vs. control (no acrolein, no COS–5 and N–5); ** *p* < 0.01 vs. acrolein.

The involvement of oxidative-stress-triggered apoptosis in retinal endothelial cells was considered as the leading cause of AMD [2,36,37]. In this study, RPE cells were stimulated by acrolein to induce oxidative stress. It was evaluated for the capacity of COS–5 and N–5 to prevent oxidative-stress-induced cell death and the imbalance of the antioxidant system. Initially, the effects of COS–5 and N–5 on acrolein-induced ROS generation (Figure S3) and MMP decline (Figure S4) in ARPE-19 cells were evaluated at different concentrations (200, 400, 800 μM). The results showed that there were no significant differences between 400 and 800 μM. Thus, all subsequent experiments were performed with the 400 μM dose. Then, intracellular and mitochondria ROS accumulation, GSH level, and GPx and SOD activities were measured (Figure 3).

ROS are natural by-products of aerobic respiration. ROS can be controlled by various cellular antioxidant compounds and enzymes, and their overproduction would lead to cell death [38]. Compared with the control, intracellular and mitochondria ROS levels were significantly increased to about 270% and 276% after acrolein exposure, respectively (Figure 3A,B). However, pretreatment with COS–5 or N–5 at the concentration of 400 μM reduced acrolein-induced ROS production significantly. GSH is one of the most important endogenous small molecule antioxidants. As shown in Figure 3C, the intracellular GSH level was decreased significantly after acrolein exposure (about 49%). Pretreatment with COS–5 or N–5 could successfully inhibit the decrease in GSH content induced by acrolein, which increased by 39% and 41% (*p* < 0.01), respectively. GSH peroxidase (GPx) and SOD activity was decreased to 30% and 55% after acrolein treatment (Figure 3D,E), respectively. The activities of antioxidant enzymes (GPx and SOD) significantly enhanced after COS–5 or N–5 treatments. These results indicated that the excellent antioxidant activity of COSs and NACOs played a critical role in protecting cells against acrolein-induced oxidative damage.

In this study, we found that chitooligosaccharides and their derivatives could protect APRE-19 cells from acrolein oxidative damage by improving their antioxidant capacities. However, without acrolein exposure, COS–5 or N–5 pretreatment did not affect these antioxidant biomarkers when compared to control cells (Figure 3). This is an interesting phenomenon. ROS at a low level play important roles as signaling molecules in normal physiology. Navdeep et al. [39] found that the mitochondrial complex III ROS was essential for T cell activation both in vitro and in vivo. It is a huge advantage that the antioxidant activities of N–5 and COS–5 were selective, and they did not affect ROS balance and ROS-mediated signaling pathways in normal cells. The data above showed that COS–5 or N–5 has the potential to be studied further and developed into a novel therapeutic agent for the treatment of AMD.

### 2.4. COS–5 and N–5 Improved Mitochondrial Function in Acrolein-Treated ARPE-19 Cells

**Figure 4:**
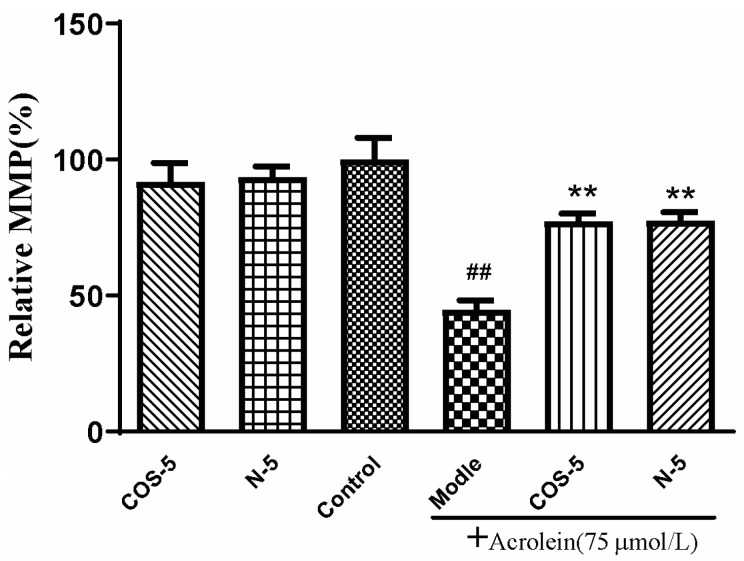
Protective effect of COS–5 and N–5 against acrolein-induced ARPE-19 mitochondrial dysfunction. The cells were pretreated with 400 μM COS–5 or N–5 for 48 h and then treated with 75 μM acrolein for an additional 24 h. The effects of COS–5 or N–5 on mitochondrial membrane potential were tested using the JC-1 method. Data are red/green (590/530 nm) fluorescence ratios. The data are expressed as ratio relative to controls. Values are mean ± SD of five separate experiments. ^##^ *p* < 0.01 vs. control (no acrolein, no COS–5 and N–5); ** *p* < 0.01 vs. acrolein.

Mitochondria are the main sites of oxidant generation, and are easily affected by oxidants, resulting in mitochondrial dysfunction and apoptosis. We examined mitochondrial function by assaying cellular and mitochondrial ROS production, and mitochondrial membrane potential MMP. The results of cellular (Figure 3A) and mitochondrial ROS production is shown in Figure 3B. MMP is an important index of mitochondrial function, which could be evaluated using a JC-1 fluorescent probe. As shown in Figure 4, MMP was decreased to about 45% by acrolein (75 μM, 24 h), which was consistent with previous reported results [32]. MMP was significantly increased after pretreatment with COS–5 or N–5. Similarly, COS–5 or N–5 did not affect mitochondrial function of normal ARPE-19 cells.

**Figure 5:**
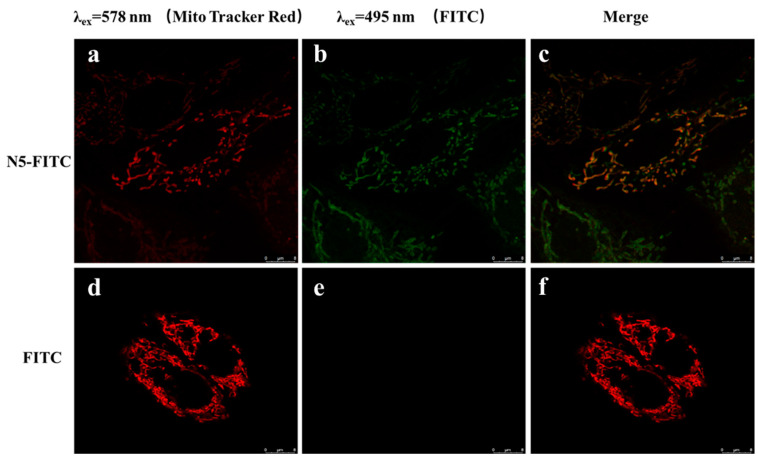
The intracellular localization of N–5 in ARPE-19 cells. The cells are stained with FITC and MitoTracker Red CMXRos. Red: MitoTracker Red CMXRos (**a**,**d**), Green: FITC (**b**,**e**), Merge images (**c**,**f**). Images were captured with confocal microscope. Scale bar: 8 μm.

Zhou [40] found that COS could entered into cells in a dose-dependent and time-dependent manner, and COS was localized preferentially in the mitochondria. However, it was not reported whether NACOSs could enter into cells. Here, the localization of N–5 in ARPE-19 cells was detected by confocal microscopy using the FITC-labeled N–5 (N5-FITC). After treatment with N5-FITC (100 μM) for 3 h, a green fluorescence was observed around the mitochondria, while nearly no fluorescence was found in control cells (Figure 5), suggesting that N–5 could enter into ARPE-19 cells and localize in the mitochondria. These data indicated that the intracellular localization of chitooligosaccharides was not affected by the introduction of acetyl group into amino. Taken together, the results demonstrated that N–5 could localize in the mitochondria and protect ARPE-19 cells against mitochondrial dysfunction and apoptosis induced by oxidative stress.

### 2.5. N–5 Promoted Nrf2 Nuclear Translocation and Increased Antioxidant Enzyme Expression

Nuclear transcription factor Nrf2 plays a key role in regulating the expression of phase II detoxification enzymes and antioxidant enzymes. Under normal physiological conditions, Nrf2 was present in the cytoplasm coupled with the negative regulatory protein Kelch Ech-associated protein 1 (Keap1), which interacted with Nrf2 and acted as an adaptor protein, maintaining Nrf2 at a low level and allowing it to be continuously degraded by the proteasome in a ubiquitin-mediated process [41]. When cells were exposed to oxidative stress, Nrf2 in the cytoplasm was released from the negative regulatory protein Keap-1 and translocated to the nucleus, then bonded to an antioxidant response element (ARE). Then, a variety of genes, including glutathione reductase (GR), heme oxygenase-1 (HO-1), catalase (CAT), NAD(P)H Quinone oxidoreductase-1 (NQO-1), and γ-glutamyl cysteine ligase (GCL) were regulated to resist the cell damage caused by oxidative stress [42].

**Figure 6:**
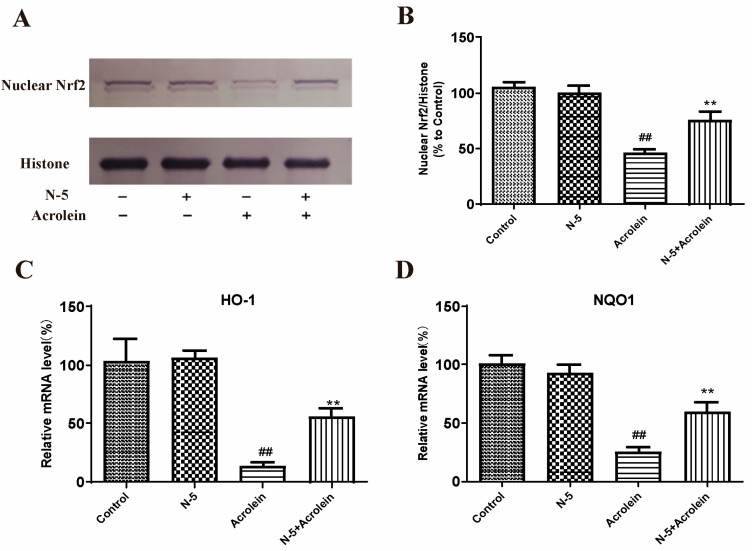
N–5 promoted Nrf2 nuclear translocation and upregulated antioxidant enzyme expressions in the ARPE-19 cell model of acrolein damage. The cells were pretreated with 400 μM N–5 for 48 h and then treated with 75 μM acrolein for an additional 24 h, and mRNA and protein levels were analyzed. Western blot image of nuclear Nrf2 (**A**) and quantification of Western blots (**B**), mRNA expression of HO-1 (**C**) and NQO1 (**D**). The data expressed as ratio relative to controls. Values are mean ± SD of three separate experiments. ^##^ *p* < 0.01 vs. control (no acrolein, no N–5); ** *p* < 0.01 vs. acrolein.

We determined the effect of oxidative stress induced by acrolein on Nrf2 nuclear translocation in the ARPE-19 cell. Due to the similar activity of N–5 and COS–5, as well as the easy preparation of N–5, we focused on N–5 in subsequent experiments. As Figure 6A,B shows, the level of Nrf2 protein in the nucleus significantly decreased after acrolein damage, similar to a published report [43], while pretreatment with N–5 significantly increased the level of nuclear Nrf2, indicating that N–5 could promote Nrf2 nuclear translocation.

Meanwhile, we detected the effect of N–5 on the transcription of genes downstream of Nrf2. The mRNA expression of HO-1 and NQO-1 were performed via qRT-PCR. As shown in Figure 6C,D, the mRNA levels of HO-1 and NQO-1 were significantly reduced in ARPE-19 cells treated with acrolein, and upregulated significantly when pretreated with N–5. These results suggested that N–5 could activate the Nrf2-ARE pathway in ARPE-19 cells, enhance Nrf2 protein nuclear translocation and upregulate the expression of phase II metabolizing enzymes (such as HO-1 and NQO1) to alleviate acrolein-induced oxidative injury.

Oxidative damage of RPE cells was a major factor in the pathogenesis of AMD, and protecting RPE from oxidative damage and death has become a trend in the treatment and prevention of AMD disease. COS and their derivatives were well-known for their free radical scavenging potential by interrupting radical chain reactions to inhibit oxidative damage [44]. The antioxidant activity of chitosan increased with decreasing Mw [45]. Li et al. [46] reported that COS had strong antioxidant activities such as hydroxyl and superoxide radical scavenging activity and reducing power. Qu [47] found that chitooligosaccharides had a certain radical scavenging activity in vitro, and they protected mice from oxidative stress, increased the activity of SOD, catalase, and GPx significantly in mice on a high-fat diet. However, there are fewer reports on *N*-acetylated oligochitosan with the same repeated unit as chitin. Several high-purity chitosan oligosaccharides and their *N*-acetylated derivatives were prepared in this study, and their protective effect on retinal pigment epithelial cells was studied. Similar to other antioxidants such as curcumin analogs [32], luteolin [48], naringenin [49], or tocopherol [31], chitooligosaccharide monomers also had good protective activity, and COS–5 and N–5 showed the best activities.

Further studies found that acetyl group introduction did not affect the protective effect of chitooligosaccharides. Subsequent study found that N–5 could enhance the antioxidant capacity of ARPE-19 cells, via reducing ROS production, increasing the GSH level, and enhancing SOD/GPx enzyme activity. In addition, N–5 could localize in mitochondria, increase MMP, reduce mitochondrial dysfunction and cellular damage, and enhance Nrf2 nuclear translocation and the transcription of downstream antioxidant enzyme (HO-1 and NQO1). Interestingly, N–5-mediated antioxidant properties were selective and associated with the oxidative stress state. N–5 does not inhibit ROS production and ROS-mediated signaling pathways in the normal cells. The above results indicated that *N*-acetylated chitooligosaccharides may have a potential application in anti-AMD degenerative diseases.

## 3. Materials and Methods

### 3.1. Materials

Chitosan (deacetylation > 95%) was purchased from Jinhu Crust Product Corp (zi bo, Shandong, China). Chitosanase fermented by Renibacter ium sp.QD1 was obtained from the Ocean University of China. Acrolein was purchased from Xiya Reagent (Chengdu, China). MitoTracker Red CM-H_2_Xros and Trizol Reagent were purchased from Invitrogen (Foster City, CA, USA). PrimeScript RT-PCR Kit was purchased from TaKaRa (Dalian, China). The reduced glutathione (GSH) assay kit was purchased from the Nanjing Jiancheng Bioengineering Institute (Nanjing, China). The MTT cell proliferation and cytotoxicity detection kits, phenyl methane sulfonyl fluoride (PMSF), reactive oxygen species (ROS) detection kit, mitochondrial membrane potential (MMP) detection kit, BCA protein assay kit, CuZn/Mn-SOD assay kit (WST-8), cellular glutathione peroxidase (GPx) assay kit, Nuclear and Cytoplasmic Protein Extraction kit, PVDF membranes, and BCIP/NBT Alkaline Phosphatase Color Development kit were purchased from the Beyotime Institute of Biotechnology (Shanghai, China). Nrf2 XP Rabbit mAb and Histone H3 XP Rabbit mAb were purchased from Cell signaling technology (Boston, MA, USA). All other reagents were obtained from Sigma-Aldrich (Saint Louis, MO, USA), unless otherwise stated.

### 3.2. Chitosan Oligosaccharide (COSs) Preparation and Purification

The COSs were prepared via the enzymatic hydrolysis of chitosan and purified with gel filtration chromatography according to a previously reported method [24]. In brief, chitosan (10 g) was added to 80 mL of distilled water, then 1.5 mL of chitosanase solution (10 U/mL) was added. The mixture was stirred at 50 °C for 24 h, and the pH of the reaction mixture was adjusted to 5~6 with HCl solution (4 mol/L) during the hydrolysis process. The hydrolysate was adjusted to pH 8~9 with NaOH solution (1 mol/L) and filtered to remove insoluble parts. The filtrate was concentrated and precipitated by adding a four-fold volume of ethanol at 4 °C overnight. The precipitate was collected via centrifugation for 15 min at 8000 rpm, and then lyophilized to yield powdered products, and identified as a COS mixture.

The COS mixture (200 mg) was dissolved in 2 mL of 0.1 M NH_4_HCO_3_, and then filtered with a microporous membrane (0.22 μm) to obtain a clear solution. The filtrate was loaded on a Bio Gel P6 column (2.6 × 110 cm) that was connected to an AKTA UPC100 purification system (GE Healthcare, Fairfield, CT, USA) equipped with an online refractive index detector. The column was eluted with 0.1 M NH_4_HCO_3_ solution at a flow rate 0.5 mL/min. Eluents (8 mL/tube) were collected using a fraction collector to afford the pure dimers, trimers, tetramers, pentamers, and hexamers of the COSs. The COSs were analyzed using the high-performance liquid chromatography (HPLC), mass spectra, nuclear magnetic resonance (NMR) and Fourier-transform infrared spectroscopy (FT-IR) methods [24].

### 3.3. N-Acetylated Chitooligosaccharide (NACOs) Preparation and Purification

The NACOs were prepared via the acetylation of COSs according to a previously reported method [26]. Briefly, the dried COS mixture (1 g) and NaHCO_3_ (756 mg) were added to methanol–water solution (8:1; *v*/*v*, 35 mL) with stirring, and 5 mL of acetic anhydride was added dropwise at 0 °C with stirring. After stirring for 4 h at room temperature, the NACO mixture solution was filtered to remove insoluble parts and the reaction completion was monitored using TLC (*n*-propanol:water, 2:1, *v*/*v*). The filtrate was concentrated and lyophilized to obtain the NACO mixture powder.

Then, the NACO mixture (500 mg) was dissolved in 2 mL of water, and filtered with a microporous membrane (0.22 μm). The filtrate was loaded on a graphitization of carbon black column (2.6 × 20 cm) that was connected to an AKTA UPC100 purification system (GE Healthcare, Fairfield, CT, USA). After loading the sample, the column was eluted with the following gradient of water and ethanol with a gradient of solvent B (ethanol) as follows: 0% B for 3 CV (column volume), then up to 60% B over 5 CV. Eluents (10 mL/tube) were collected using a fraction collector and monitored using TLC (*n*-propanol:water, 2:1, *v*/*v*). Pure dimers, trimers, tetramers, pentamers, and hexamers of the NACOs were pooled and lyophilized. The NACO samples were identified via HPLC chromatogram, mass spectra, nuclear magnetic resonance (NMR) and Fourier-transform infrared spectroscopy (FT-IR) analysis.

### 3.4. MTT Assay for Cell Viability

The ARPE-19 (human retinal pigment epithelial) cell line was purchased from ATCC (CRL2302) and cultured in a DMEM-F12 medium supplemented with 10% fetal bovine serum, 0.348% sodium bicarbonate, 2 mM L-glutamine, 100 μg/mL of streptomycin, and 100 U/mL of penicillin. The cell culture was maintained at 37 °C in a humidified atmosphere of 95% air and 5% CO_2_ [50]. ARPE-19 cells were used within 10 generations, and the medium was changed every two days. COSs and NCOSs were dissolved with PBS buffer, filtered through a sterile 0.22 μm filter, and diluted with complete culture medium to different concentrations for the cell experiments.

The ARPE-19 cells were seeded in 96-well plates at 5 × 10^4^ cells per well and incubated overnight. After incubation with different concentrations of COSs or NCOSs for 48 h, the cells were treated with 75 μM acrolein for 24 h. Cell viability was measured via MTT cell proliferation and a cytotoxicity detection kit (Beyotime). After 4 h of incubation with MTT, the solubilization buffer was added to each well and incubated at 37 °C overnight. The optical densities were read at 555 nm using a SpectraMax M5 plate reader (Molecular Devices, Sunnyvale, CA, USA).

### 3.5. Antioxidant Enzyme Activities, ROS Generation, and Intracellular GSH Levels Assay

The GSH level, superoxide dismutase (SOD) activity, and GPx activity were determined using commercial assay kits [51]. Briefly, cells were placed in 6-well plates at a density of 5 × 10^5^ cells per well. After 12 h, the cells were treated for 48 h with 400 μM of COS–5 or N–5 and then for 24 h with or without 75 μM acrolein. After treatment, the cells were washed twice with PBS, and then the antioxidant enzyme activities and GSH level in the cells were detected.

Moreover, the ROS levels in PRE cells and mitochondria exposed to acrolein were determined using fluorescent probe. In brief, cells were plated in 96-well plates at a density of 2.5 × 10^4^ cells per well for 12 h. ARPE-19 cells were treated with 400 μM of COS–5 or N–5 for 48 h, and then incubated with or without 75 μM acrolein for another 24 h. The ROS level in PRE cells was determined by the 2′, 7′-dichlorofluorescein diacetate (DCFH-DA) method using a SpectraMax M5 plate reader (Molecular Devices, San Jose, CA, USA) at a 488 nm excitation wavelength and a 525 nm emission wavelength [52]. The ROS generation in mitochondria was detected using MitoTracker Red CM-H_2_Xros at a 579 nm excitation wavelength and a 599 nm emission wavelength.

### 3.6. Confocal Imaging

ARPE-19 cells were cultured on glass-bottom cell culture dishes at a density of 2 × 10^4^ cells per well for 12 h. The cells were incubated with 25 nM MitoTracker Red CMXROS at 37 °C for 30 min. Thereafter, the cells washed three times with PBS to remove unbound probes. Then, the cells were incubated with FITC (100 μM) or FITC-labeled N–5 (100 μM) for 3 h at 37 °C. Cellular uptake was terminated by washing the cells three times with PBS. Finally, the cells were observed under a Nikon A1 confocal microscope (Nikon Corporation, Tokyo, Japan). The green fluorescence of FITC was measured at Ex495/Em525, and the red fluorescence of MitoTracker Red CMXRos was measured at Ex578/Em599 [53].

### 3.7. Mitochondrial Dysfunction Evaluation

Mitochondrial membrane potential (MMP) was detected in live ARPE-19 cells using a cationic fluorescent indicator JC-1, according to the manufacturer’s instructions. Briefly, APRE-19 cells were seeded at a density of 2.5 × 10^4^ cells per well in a 96-well plate. After 12 h, the cells were exposed to 400 μmol/mL of N–5 for 48 h. After treatment with 75 μmol/mL of acrolein for 24 h, the cells were treated with JC-1 for 30 min at 37 °C, washed with PBS, and observed under the fluorescence microscope. The Δψm of ARPE-19 cells in each treatment group was calculated as the fluorescence ratio (590 to 530 nm) [54].

### 3.8. Western Blot

Western blot was performed as in previously described methods [55] and each Western blot was repeated at least three times. Nuclear proteins were prepared using a Nuclear and Cytoplasmic Protein Extraction Kit, and nuclear Nrf2 was analyzed using Western blot. Briefly, the lysates were homogenized and centrifuged at 13,000 ×g for 15 min at 4 °C. The supernatants were collected, and the protein concentrations were determined using the BCA Protein Assay kit. Equal amounts (20 μg) of each protein sample were loaded on 10% SDS-PAGE gels, electrophoresed, transferred to PVDF membranes, and blocked with 5% non-fat milk. The membranes were incubated with anti-Nrf2 (1:1000) and anti-histone H3 (1:1000) at 4 °C overnight, and then incubated with anti-mouse secondary antibodies at room temperature for 1 h. Protein bands were visualized using a BCIP/NBT Alkaline Phosphatase Color Development Kit. Signals were quantified using ImageJ software (Version 1.52b, NIH, Baltimore, MD, USA), and defined as the ratio of target protein to histone H3.

### 3.9. Real-Time PCR

Real-time PCR was performed using a previously described method [56]. Total RNA was extracted from the cells using Trizol reagent according to the manufacturer’s protocol. Reverse transcription was performed using the PrimeScript RT-PCR Kit followed by semiquantitative real-time PCR using specific primers. The primer sequences are listed in Table 3.

### 3.10. Statistical Analysis

All quantitative experiments were repeated at least 3 times independently. Data are presented as mean ± SD. Data were analyzed by one-way analysis of variance (ANOVA) with Tukey’s multiple comparison post hoc test using GraphPad Prism 8.0 Statistics Software (Graphpad Software, Inc., La Jolla, CA, USA). A *p* value of < 0.05 was considered statistically significant.

## 4. Conclusions

In conclusion, our study demonstrated that chitosan oligosaccharides (COSs) and their *N*-acetylated chitooligosaccharides (NACOs) exhibited excellent protection effects on acrolein-induced ARPE-19 cell damage. Among the monomers, COS–5 or N–5 pretreatment significantly reduced reactive oxygen species production, raised the intracellular level of GSH and the activity of SOD and GSH-Px, and attenuated the loss of mitochondrial membrane potential. Further study indicated that the N–5 could localize in the mitochondria and promote Nrf2 nuclear transfer and the expression of downstream phase II detoxification enzymes. These results suggest that COSs and NACOs might be promising antagonists against acrolein-induced APRE-19 cell death.

## Figures and Tables

**Table 1:** The MS data of COS and NACO samples.

	Molecular Formula	Mw	*m*/*z*
COS–2	C_12_H_24_N_2_O_9_	340.1	[M + H]^+^ = 341.2 [M + Na]^+^ = 363.2
COS–3	C_18_H_35_N_3_O_13_	501.2	[M + H]^+^ = 502.3 [2M + H]^+^ = 1003.7
COS–4	C_24_H_46_N_4_O_17_	662.3	[M + H]^+^ = 663.4
COS–5	C_30_H_57_N_5_O_21_	823.4	[M + H]^+^ = 824.6 [M + 2H]^+^ = 412.8
COS–6	C_36_H_68_N_6_O_25_	984.5	[M + H]^+^ = 985.9 [M + 2H]^+^ = 493.5
N–2	C_16_H_28_N_2_O_11_	424.4	[M + H]^+^ = 425.2
			[M + Na]^+^ = 447.2
N–3	C_24_H_41_N_3_O_16_	627.6	[M + H]^+^ = 628.3
			[2M + H]^+^ = 1255.5
N–4	C_32_H_54_N_4_O_21_	830.8	[M + H]^+^ = 831.3
N–5	C_40_H_67_N_5_O_26_	1033.9	[M + H]^+^ = 1034.4
N–6	C_48_H_80_N_6_O_31_	1237.1	[M + H]^+^ = 1237.8

**Table 2:** The ^13^C NMR date of COS–3 and N–3.

Sample		NMR Data (ppm)							
		C=O	CH3	C1	C2	C3	C4	C5	C6
COS–3	GlcN″			100.4	58.5	74.4	72.3	79.1	62.9
	GlcN′			100.2	58.5	79.1	77.4	72.8	62.7
	GlcNβ			95.3	59.3	70.6	79.1	72.6	62.7
	GlcNα			91.6	56.9	70.6	79.1	72.6	62.7
N–3	GlcNAc″	174.8	22.6 22.5 22.3	101.8	56.0	73.9	70.1	76.3	61.0
	GlcNAc′			101.6	55.4	72.6	79.6	74.9	60.4
	GlcNAcβ			95.2	56.5	72.9	79.6	75.0	60.5
	GlcNAcα			90.8	54.1	69.6	80.1	70.4	60.4

**Table 3:** Primer sequences.

Primers	Forward	Reverse
HO-1	GGTCCTTACACTCAGCTTTCT	CATAGGCTCCTTCCTCCTTTC
NQO1	AAAGGACCCTTCCGGAGTAA	CCATCCTTCCAGGATTTGAA
β-actin	ACCCTGAAGTACCCCATCGAG	GGATAGCACAGCCTGGATAGCA

## Data Availability

Not applicable.

## Supplementary Materials

The following supporting information can be downloaded at: https://www.mdpi.com/article/10.3390/md21030137/s1, Figure S1: The cytotoxicity of COSs, NACOs and PACOs in ARPE-19 cell; Figure S2: Effects of COSs and NACOs on the proliferation of ARPE-19 cells; Figure S3: COS–5 and N–5 against acrolein-induced oxidative stress; Figure S4: Protective effect of COS–5 and N–5 against acrolein-induced ARPE-19 mitochondrial dysfunction.
