# Supplementary material for: Chitooligosaccharides Derivatives Protect ARPE-19 Cells against Acrolein-Induced Oxidative Injury
Source: Mar Drugs. 2023 Feb 22;21(3):137. doi: 10.3390/md21030137 (PMC10058944; doi:10.3390/md21030137)
Supplement: Supplementary file 1 [file marinedrugs-21-00137-s001.zip › marinedrugs-2218960-supplementary.pdf]

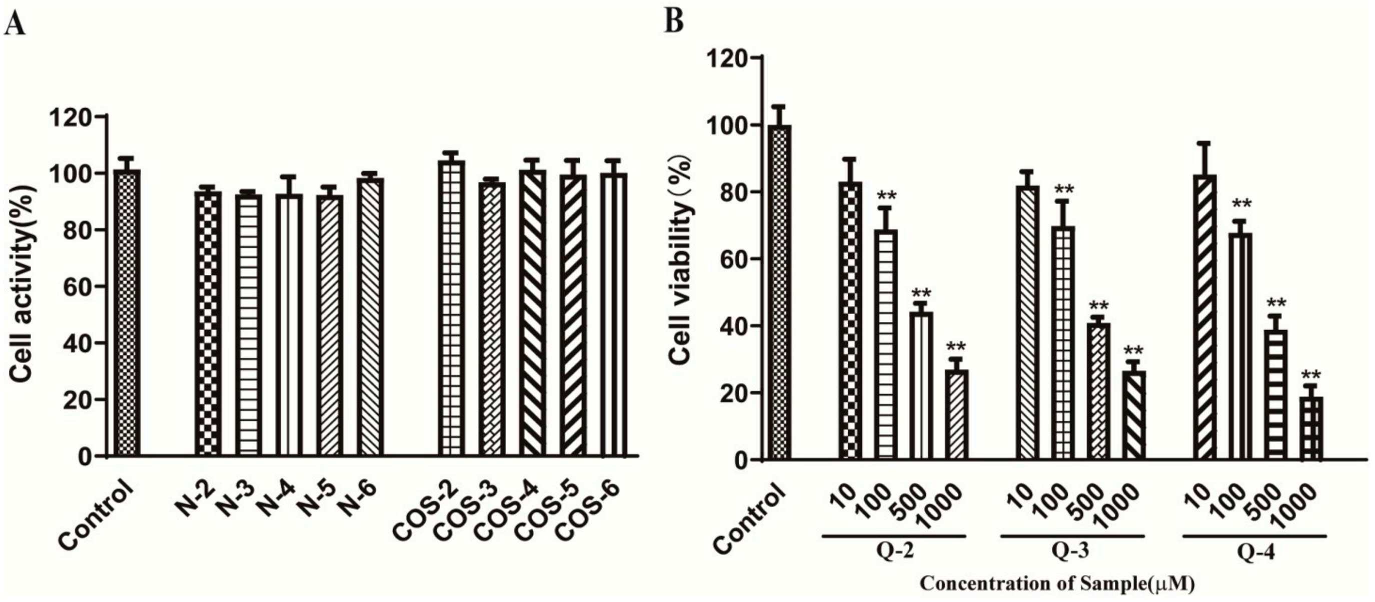

Figure S1. The cytotoxicity of COSs, NACOs and PACOs in ARPE-19 cell. The cells were treated with 1 mM COSs or NACOs (A) or 10, 100, 500, 1000 μM PACOs (B) for 24 h. Cell viability was analyzed using the MTT method. Values are mean ± SD of five separate experiments. ***p* < 0.01 vs. control.

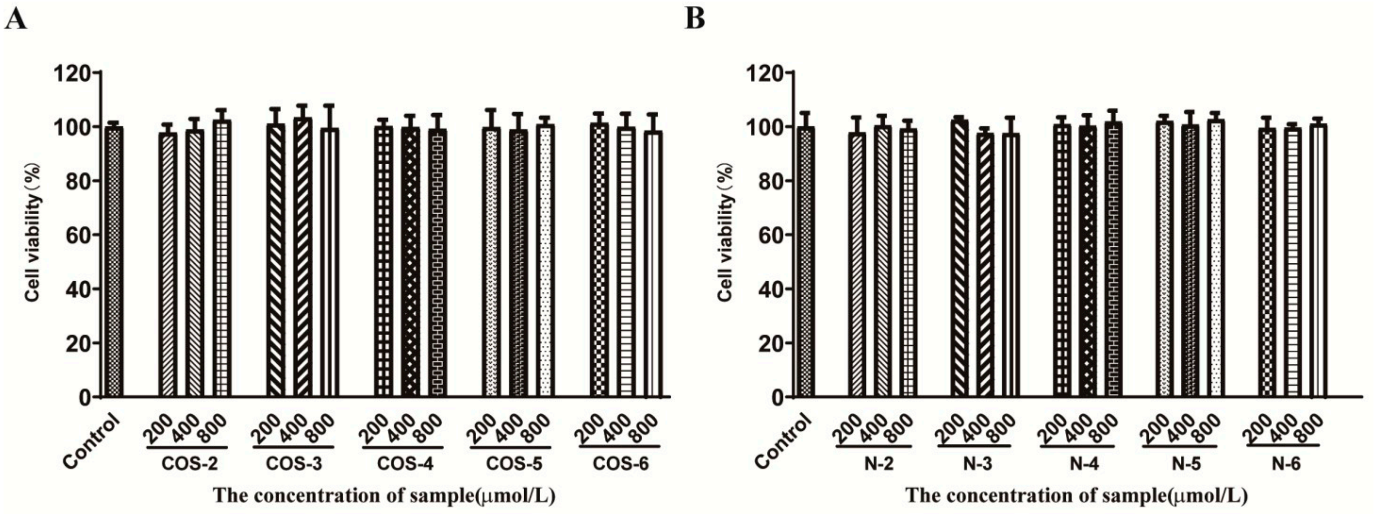

Figure S2. Effects of COSs and NACOs on the proliferation of ARPE-19 cells. The cells were treated with 200, 400, 800 μM COSs (A) or NACOs (B) for 48 h. Cell viability was analyzed using the MTT method. Values are mean ± SD of five separate experiments.

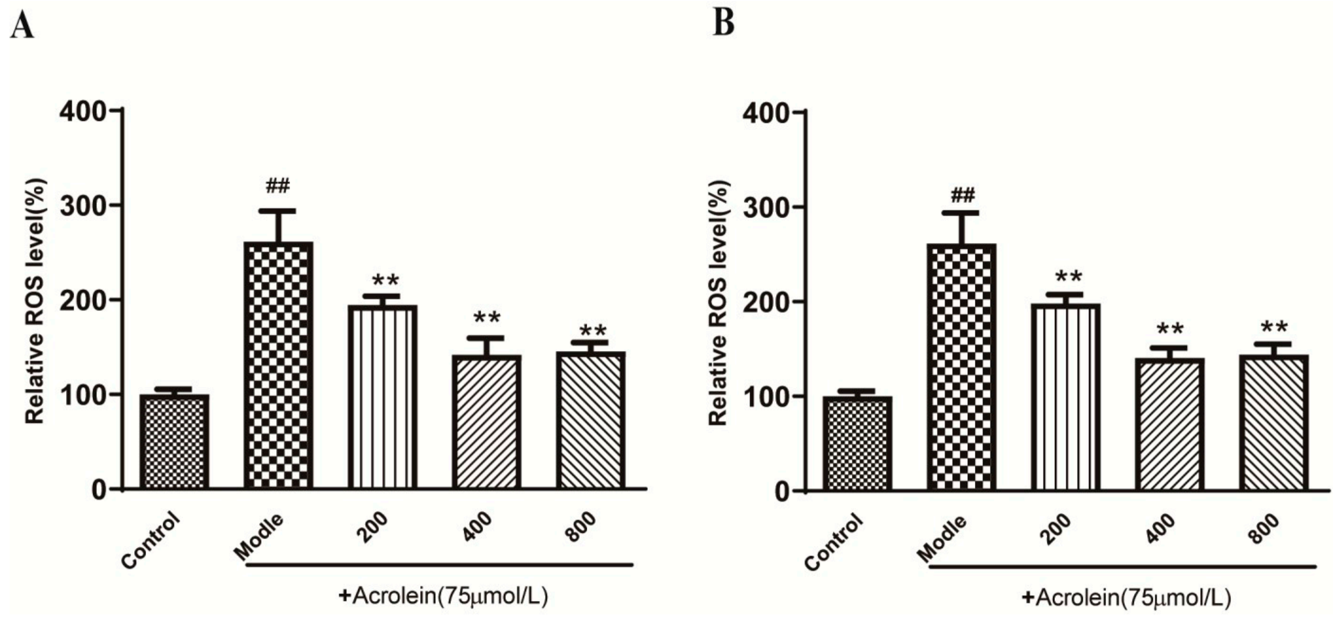

Figure S3. COS-5 and **N-5** against acrolein-induced oxidative stress. ARPE-19 cells were treated with 200, 400, 800 μM **COS-5** (A) or **N-5** (B) for 48 h and then treated with acrolein for additional 24 h. Cellular ROS generation in PRE cells was determined by the 2′, 7′-dichlorofluorescein diacetate (DCFH-DA) method. The data expressed as ratio relative to controls. Values are mean ± SD of three separate experiments. ##*p* <0.01 vs. control (no acrolein, no **COS-5** and **N-5**); **p* <0.05, ***p* < 0.01 vs. acrolein.

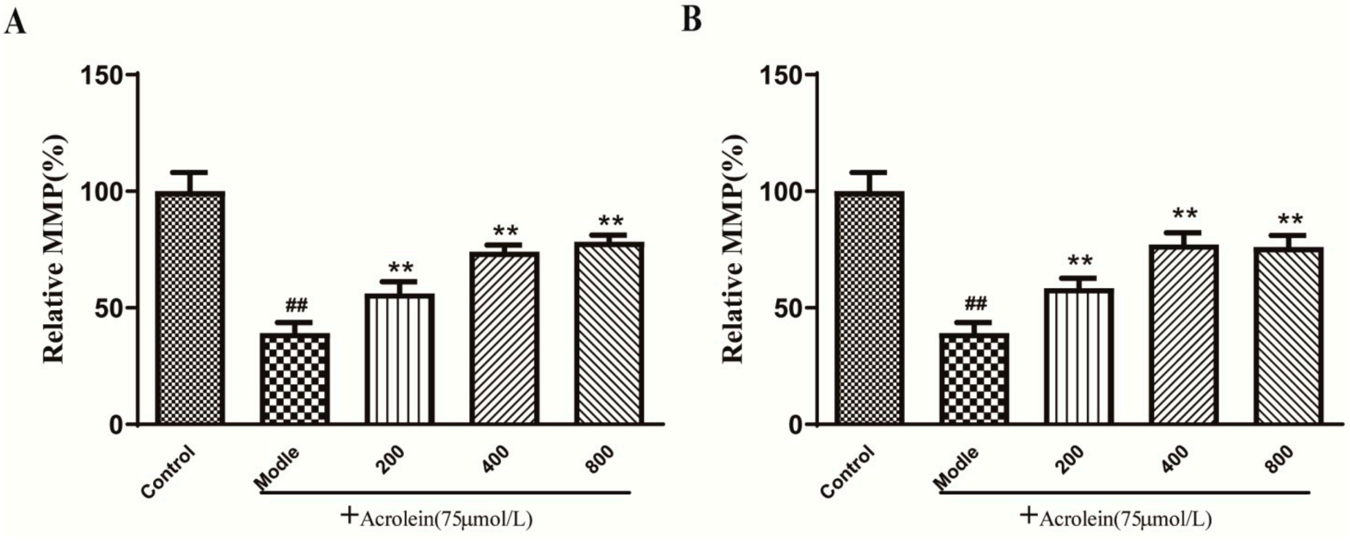

Figure S4. Protective effect of **COS-5** and **N-5** against acrolein-induced ARPE-19 mitochondrial dysfunction. The cells were pre-treated with 200, 400, 800 μM **COS-5** (A) or **N-5** (B) for 48 h and then treated with 75 μM acrolein for additional 24 h. The effects of **COS-5** or **N-5** on mitochondrial membrane potential were tested using the JC-1 method. Data are red/green (590/530 nm) fluorescence ratios. The data expressed as ratio relative to controls. Values are mean ± SD of three separate experiments. ##*p* <0.01 vs. control (no acrolein, no **COS-5** and **N-5**); **p* <0.05, ***p* < 0.01 vs. acrolein.
